# Anti-*Escherichia coli* Functionalized Silver-Doped Carbon Nanofibers for Capture of *E. coli* in Microfluidic Systems

**DOI:** 10.3390/polym12051117

**Published:** 2020-05-13

**Authors:** Soshana Smith, Michael Delaney, Margaret Frey

**Affiliations:** 1 Department of Fiber Science and Apparel Design, Cornell University, Ithaca, NY 14853, USA; margaret.frey@cornell.edu; 2 Robert Frederick Smith School of Chemical & Biomolecular Engineering, Cornell University, Ithaca, NY 14853, USA; md839@cornell.edu

**Keywords:** functional materials via electrospinning method, microfluidics, *Escherichia coli*, *Staphylococcus aureus*, silver doped, selective capture

## Abstract

Silver-doped carbon nanofibers (SDCNF) are used as the base material for the selective capture of *Escherichia coli* in microfluidic systems. Fibers were spun in a glovebox with dry atmosphere maintained by forced dry air pumped through the closed environment. This affected the evaporation rate of the solvent during the electrospinning process and the distribution of silver particles within the fiber. Antibodies are immobilized on the surface of the silver-doped polyacrylonitrile (PAN) based carbon nanofibers via a three-step process. The negatively charged silver particles present on the surface of the nanofibers provide suitable sites for positively charged biotinylated poly-(L)-lysine-graft-poly-ethylene-glycol (PLL-*g*-PEG biotin) conjugate attachment. Streptavidin and a biotinylated anti-*E. coli* antibody were then added to create anti-*E. coli* surface functionalized (AESF) nanofibers. Functionalized fibers were able to immobilize up to 130 times the amount of *E. coli* on the fiber surface compared to neat silver doped fibers. Confocal images show *E. coli* remains immobilized on fiber mat surface after extensive rinsing showing the bacteria is not simply a result of non-specific binding. To demonstrate selectivity and functionalization with both gram negative and gram-positive antibodies, anti-*Staphylococcus aureus* surface functionalized (ASSF) nanofibers were also prepared. Experiments with AESF performed with *Staphylococcus aureus* (*S. aureus*) and ASSF with *E. coli* show negligible binding to the fiber surface showing the selectivity of the functionalized membranes. This surface functionalization can be done with a variety of antibodies for tunable selective pathogen capture.

## 1. Introduction

Within the next 30 years, the world’s population is expected to reach 9 Billion people, requiring the world’s food supply to increase by more than 70% [1,2,3]. With the increase in world food production and supply comes the challenge of monitoring and detecting food borne illnesses. Currently, approximately 48 million people become ill from foodborne illnesses annually, resulting in 3000 deaths [4]. It is estimated that 95% of these cases of food borne illnesses are caused by only 15 pathogens including *Salmonella*, *E. coli, S. aureus* and others [5,6,7]. Methods for detecting these pathogens can be slow and complicated including culture-based methods and polymerase chain reactions (PCR) [8,9,10]. Recent techniques such as ELISA and biosensors allow for more rapid detection of the pathogens [11,12].

Recently, research toward biosensors has begun taking advantage of nanostructures such as nanowires and nanofibers in microfluidics for their use in the detection and capture of various pathogens [13,14,15]. Microfluidics are an attractive method for testing because of the need for a small sample volume, low cost, high throughput, and high sensitivity of detection [7]. Nanostructures, especially electrospun nanofibers, are used because of their high surface to volume ratio, easy fabrication, ease of surface modification and numerous sites for ligand immobilization. Researchers have been able to use a wide variety of materials as the basis for their nanostructures including silicon nanorods, TiO_2_, MnO_2_, PVA, carbon nanofibers [16,17,18]. 

Yu et al. and Hsaio et al. utilized both nanorods and nanofibers as the basis for their immobilization of anti-epithelial cell adhesion molecule (anti-EpCAM) positive antibodies onto the surface of their membranes using a three-step technique [19,20]. Both works were able to demonstrate capture and release EpCAM positive cancer tumor cells (CTC) with high selectivity. Zhang et al. used TiO_2_ membranes made from electrospinning titanium *n*-butoxide (TBT)/polyvinyl pyrrolidone (PVP). Fibers were further functionalized with streptavidin and anti-EpCAM antibodies to once again produce a nanofibrous membrane to successfully capture EpCAM from artificial CTC blood samples, as well as from whole blood samples. Researchers have also been able to immobilize antibodies directly onto PEI based fibers taking advantage of the –NH_2_ groups present [21,22]. Similarly *N*-hydroxysuccinimide (NHS) chemistry was utilized to activate the carboxylic acid groups on the surface of the PLGA nanofibers in order affix anti-CD146 antibodies onto the surface [23].

In this work, we aim to selectively capture *E. coli* in microfluidic channels using the antibody-antigen binding. Currently there is limited research taking advantage of this known interaction for capturing bacteria. Jin et al. incorporated the anti-*E. coli* O157 antibody directly into the PVA solution [24]. The water-soluble fibers immediately dissolved when it came into contact with *E. coli* containing inlet stream allowing the antibody to interact with the antigen. However, this method does not provide a suitable way to remove the bacteria from the solution. A non-water-soluble polymer could be used in the place of PVA, but a large proportion of expensive antibody would remain trapped within the fiber. Other methods use a complicated primary antibody-bacteria-secondary antibody sandwich complex [25]. The *E. coli* O157:H7 bacterium is first bound to a conductive magnetic nanoparticle while the nanofiber mat was modified with an antibody using glutaraldehyde as crosslinker. The modified bacterium solution was then passed over the fiber mat resulting in the sandwich complex that was used for their biosensor. We aim to produce a simpler process using antibodies to immobilize *E. coli* onto the fiber surface. Silver has long been used for its antibacterial properties with numerous researcher incorporating silver into nanofiber matrices and observing excellent inhibition of bacterial growth [26,27,28]. Song et al. noted that silver particles were able to maintain their antimicrobial tendencies during the carbonization process as silver nitrate reduced to silver nanoparticles [29]. In this work, silver is used for a different purpose. Taking advantage of its biocidal tendencies and the tunable surface change, researchers aim to increase the amount of bacteria that can be captured on the substrate surface by utilizing antibodies. Using silver nanoparticles as the base of our functionalization, we tether antibodies onto the surface using a biotin-streptavidin complex. Surface functionalized fibers will able to selectively capture needed pathogen with high efficiency. 

## 2. Experimental

### 2.1. Membrane Preparation

The non-woven mat was prepared using the electrospinning method. The electrospinning solution was prepared by combining 0.6 mg of polyacrylonitrile (PAN, M_W_ = 150,000, MilliporeSigma, St. Louis, MO, USA) with 0.2 mg of silver nitrate (AgNO_3_, Honeywell) and *N*,*N*-Dimethylformamide (DMF, MilliporeSigma, St. Louis, MO, USA) to form a 12 wt.% solution. The solution was left to stir overnight at room temperature. The PAN/AgNO_3_ solution was electrospun for 4 h at a flow rate of 0.25 mL/h using a 21-gauge needle. A 17.5 kV voltage was applied and 16 cm distance between the needle and the collector plate was used for all samples. Fibers were spun in a glove box with dry air being forced through the chamber as the membrane spins. The resultant fiber mat was then stabilized at 250 °C for 2 h at a ramp rate of 5 °C/min under air. Fiber mats were then carbonized at 850 °C for 2 h at a ramp rate of 5 °C/min under nitrogen gas. After carbonization, fiber mats were cut into rectangular shapes (3 cm × 0.4 cm × 15 mm) to be placed in channels.

### 2.2. Membrane Characterization

Fiber morphology and diameter were examined using scanning electron microscopy (Zeiss Gemini 500 SEM, Jena, Germany) with an accelerating voltage of 1 KV. The average fiber diameters were determined by choosing 50 randomly selected fibers using Image J 1.49v.

### 2.3. Surface Modification of Silver-Doped Carbon Nanofibers

**Figure 1:**
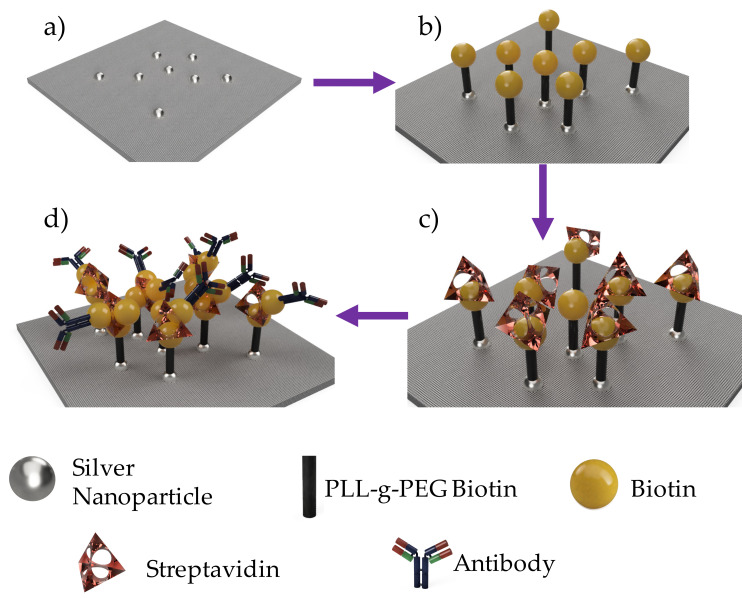
Schematic of surface functionalization steps to immobilize antibody onto nanofiber surface.

The fiber mat was first treated with PLL(20)-*g*-[3.5]-PEG(2)/PEG(3.4)-biotin(50%) (PLL-*g*-PEG-biotin) (SuSoS AG, Dübendorf, Switzerland) [300 μg·mL^−1^ in 10 mM HEPES buffer (pH 7.4)] for one hour at room temperature. Fibers are then transferred to a streptavidin (Dynabeads^TM^, Invitrogen, Waltham, MA, USA) solution [10 μg·mL^−1^ cell-culture grade 1× PBS] and incubated for 1 h. Fibers are finally immersed in either a biotinylated anti-*E. coli* (ab20640, Abcam, Cambridge, MA, USA) solution or a biotinylated anti-*S. aureus* (ab35192, Abcam, Cambridge, MA, USA) [10 μg·mL^−1^ in 1× PBS containing 1% BSA). Fibers were left in this solution for an hour and then rinsed 3 times with PBS to remove excess material. The functionalization steps are shown in a schematic in Figure 1. 

### 2.4. Colorimetric Assay

A colorimetric assay was used to determine the surface available biotin after the PLL-*g*-PEG biotin step. The assay kit used was the Pierce™ Biotin Quantitation Kit from Thermo Fisher Scientific, Waltham, MA, USA. The 4-Hydroxyazobenzene-2-carboxylic acid (HABA)/avidin solution was reconstituted in 100 μL of ultra-pure water and then added to 800 μL of 1× PBS buffer solution. First, the absorbance of the HABA/avidin solution in PBS Buffer was measured at 500 nm using a Lambda 35 UV/Vis Spectrophotometer from Perkin Elmer, Waltham, MA, USA. A pre weighed piece of fiber mat was then placed in the cuvette. The cuvette was then tipped back and forth twice then left in the HABA solution for 1 h. The fiber mat was then removed, and the absorbance of the solution was measured repeated again a 500 nm. The surface-available biotin was calculated using the following equation [30,31]:

(1) $$\mathrm{Surface}\;\mathrm{Available}\;\mathrm{Biotin}\;\left(\frac{\mathrm{mg}\;\mathrm{Biotin}}{g\;\mathrm{fiber}}\right)=\left(A_{500}^{0}-A_{500}\right)\left(\frac{{\mathrm{MW}}_{\mathrm{Biotin}}V}{\varepsilon \mathrm{bW}}\right)\times 10^{3}$$

where $A_{500}^{0}\;$ is the absorbance of the solution prior to the addition of nanofiber mat; A_500_ is the absorbance of the solution after reaction with nanofiber mat; MW_Biotin_ is the molecular weight of the biotin (244.3 g/mol); V is the volume of the solution (L); b is the cuvette path length (1 cm); ε is the extinction coefficient of the HABA/avidin complex at 500 nm (3.4 × 10^3^ L/(mol·cm)); W is fiber mat weight (g).

### 2.5. Microfluidic Channel Assembly

**Figure 2:**
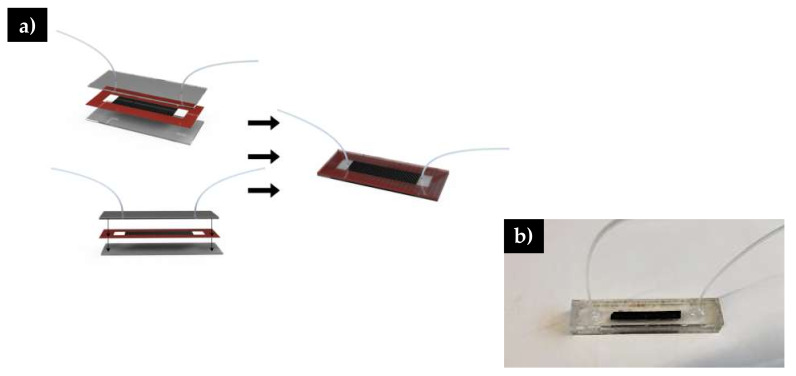
**a**) Schematic of microfluidic channel assembly. Double sided tape is highlighted in red **b**) Image of fully assembled microfluidic system.

Microfluidic channels were manufactured using a technique mentioned by previous researchers [32,33,34]. Channel parts, including flow paths and inlets/outlets, were first designed in Adobe Illustrator. The top and bottom segments of the channels (Figure 2) were then cut out of acrylic glass using a CO_2_ laser cutter. To make the channel that will house the fibers, double-sided tape was affixed to a piece of acrylic and a laser cutter was used to etch the desired pattern design into the 0.24 mm thick double-sided tape. The channel dimensions used were 6 cm × 0.5 cm. To fully assemble the channels containing the functionalized fibers, one side of the double-sided tape was attached to the bottom segment, the fiber was then placed in the channel and the top plate was affixed. After channel assembly, polyvinyl chloride tubing with a 0.15 mm external diameter was glued into the inlet and outlet holes of the channels with quick dry epoxy adhesive. The channel and tubing were then blocked with 1% BSA for one hour. 

### 2.6. Bacteria Preparation and Retention.

Fluorescent tagged bacteria *E. coli* (E2861, Invitrogen, Waltham, MA, USA) and *S. aureus* (S23372, Invitrogen, Waltham, MA, USA) were reconstituted with sterile 1× PBS to obtain the desired concentration. The solution is vigorously vortexed at the highest setting (3 × 15 s). The solution was then sonicated 6 times for 20 s each to break up aggregates of bacteria. Diluted *E. coli* solution is loaded into a 5-mL syringe and pumped through the microfluidic channel at a rate of 10 μL/min for 100 min. Effluent from this step was collected to be analyzed later. Sterile PBS buffer is then pumped though at a rate of 50 μL/min for 1 h to remove unbounded bacteria. 

Immobilized bacteria on the fiber mat surface are imaged using Zeiss LSM710 confocal microscope. Images were taken with a water immersed 40× objective using 488 nm and 561 nm laser lines. Bacteria cell density is estimated by pipetting 5 μL of bacteria solution onto a glass slide and imaged using an Olympus fluorescence microscope with Metamorph acquisition. 

## 3. Results and Discussion

**Figure 3:**
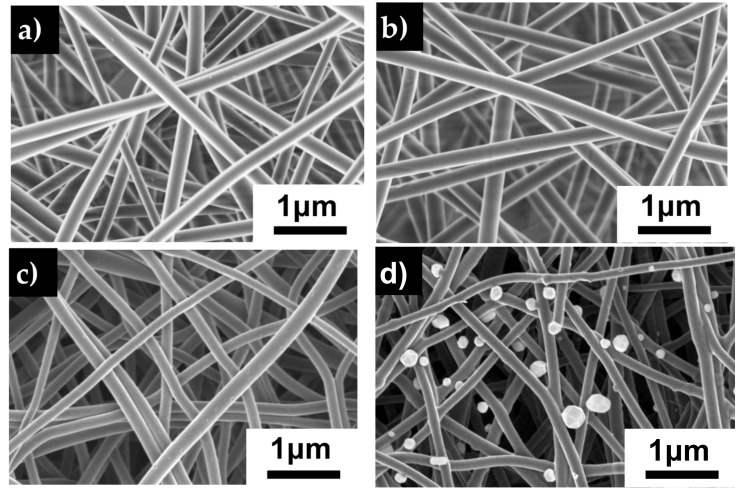

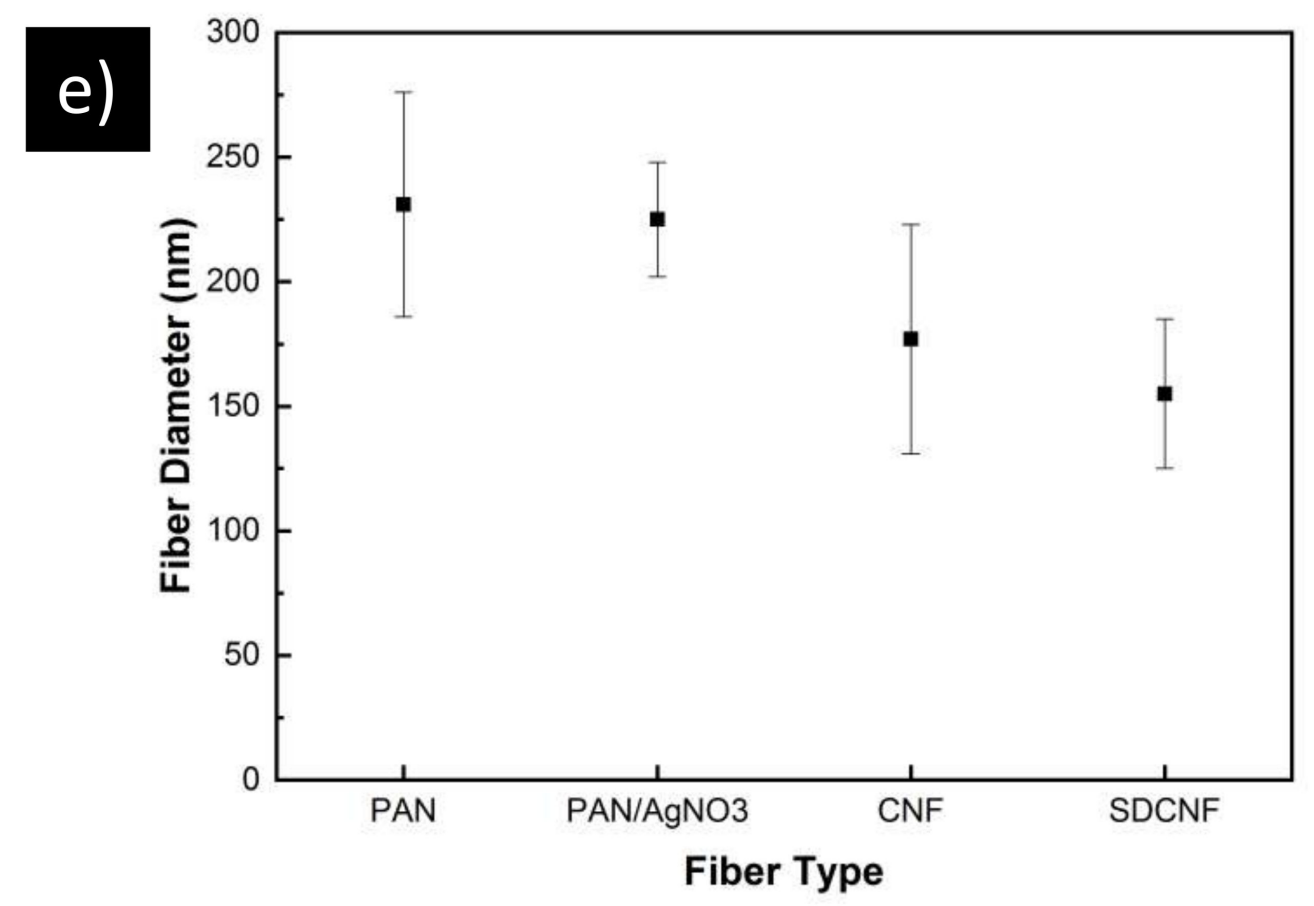
SEM images of electrospun membrane **a**) PAN **b**) PAN/AgNO_3_ **c**) carbon nanofiber **d**) silver doped carbon nanofiber. **e**) Average fiber diameters for the previously mentioned fibers. Error bars represent standard deviation for each measurement.

SEM images (Figure 3) of all fibers reveal a 3D interconnected porous membrane with randomly oriented, uniform, well-formed fibers free of beads. The PAN and PAN/AgNO_3_ fibers have a similar fiber diameter averaging 225 nm (Figure 3e). Upon carbonization, the average fiber diameter reduces to 177 ± 46 nm for CNF and 154 ± 30 nm for the CNF/Ag fibers. Figure 3d shows circular objects attached to the carbon nanofibers varying in size from 120 to 255 nm. Silver nanoparticles on the fiber surface have a polydispersity index (PDI) of 0.3, indicating an acceptable level of monodispersity of the particles [35]. EDX analysis shows that these are silver particles that have been exposed on the surface of the fibers during the carbonization process. The silver particles are a result of the thermal decomposition of the silver nitrate particles which occurs around 440 °C [36]. 

**Figure 4:**
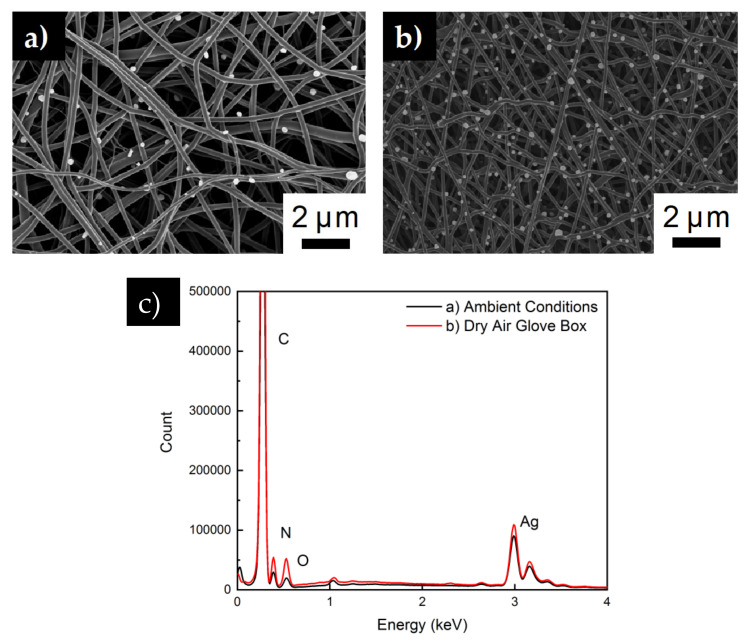
(**a**) SDCNF spun under ambient conditions (**b**) SDCNF spun in a glove box with dry air being constantly pumped through (**c**) EDX spectra of previously mentioned fibers.

In order to obtain the morphology seen in Figure 3d, the environment during the electrospinning process is observed to be significant. Usually, in-situ polyacrylonitrile-based silver-doped carbon nanofibers achieved through fiber mat calcination results in very little silver on the surface of the fibers [37]. Initially fibers spun under ambient conditions (20 °C, 20% relative humidity (RH)) result in the fibers seen in Figure 4a. In this image, there are approximately 1.3 × 10^5^ silver particles per square mm attached to carbon nanofiber surface. However, in Figure 4b, fibers spun in a forced dry air environment result in 1.5 × 10^6^/mm^2^ of silver particles on the fiber surface. EDX spectra analysis (Figure 4c) shows that the silver contents of the two membranes are very similar. Indicating the silver particles in Figure 4a are trapped inside the fiber instead of being exposed on the surface. Within the closed environment of the glovebox, the humidity is approximately 5% RH, in addition, the air in the box is continuously being replenished. Currently, there are no in-depth studies on the influence of humidity on the dispersion of nanoparticles in nanofibers. However, Barua et al. found in their work that a lower relative humidity leads to longer phase separation time within of the polymer from the solvent within the PAN fibers [38]. A longer phase separation time would give the silver nitrate particles more time to migrate near the fiber surface at lower humidity. In addition, it has also been shown that additional extensional deformation provided by air flow can lead to better dispersion of nanoparticles in electrospun fibers and lessen agglomeration towards the fiber center [39]. An increase in silver nitrate particles towards the edge of the fibers leads to more opportunity for more silver particles to be exposed as the fiber carbonizes. As Figure 3e shows, fiber diameter of the SDCNF fibers are 100nm thinner than the precursor PAN/AgNO_3_ fibers. As the fiber diameter decreases, there silver nitrate nanofibers distributed towards the edge of the fiber become exposed. 

**Figure 5:**
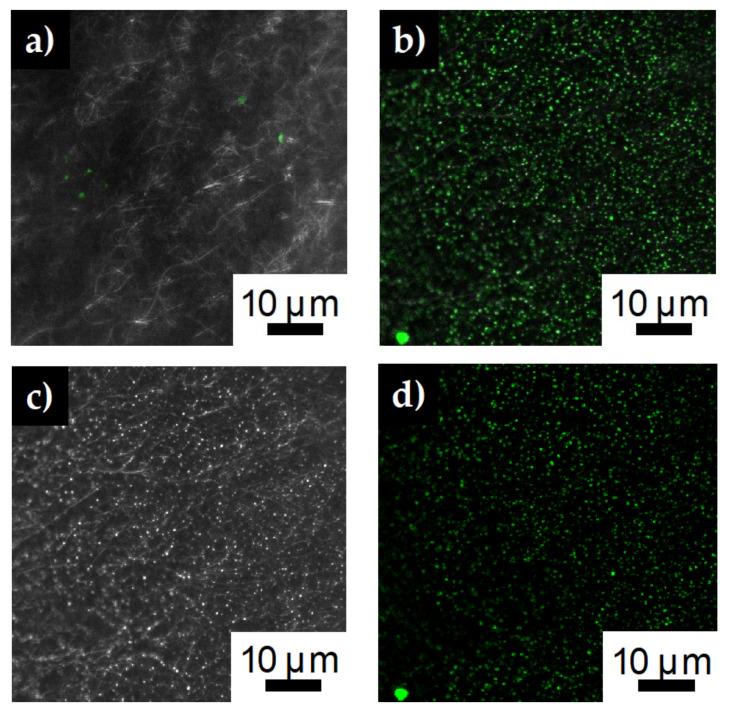
Confocal images after immersing (**a**) carbon nanofibers and (**b**) silver impregnated carbon nanofibers in PLL-*g*-PEG FITC solution for one hour. (**c**) Bright field image from confocal images showing silver nanofibers (**d**) 488 nm laser line of the confocal images showing the green fluorescence of the FITC immobilized on the fiber surface.

Both carbon nanofibers and silver doped carbon nanofibers were immersed in PLL-*g*-PEG FITC solution and then thoroughly rinsed. Confocal mages of the carbon nanofibers show little to no fluorescence indicating there was no conjugate attachment to the fibers (Figure 5a). However, the silver impregnated fibers show discrete areas of fluorescence (Figure 5b). Splitting the composite confocal image into the 488 nm laser line (Figure 5d) and the bright field image (Figure 5c), shows the areas of green fluorescence corresponds to the brighter areas of the bright field image. The brighter areas of the bright field image correspond to silver particles on the surface of the fiber membrane referenced in the SEM images (Figure 3d). This result strongly indicates the PLL-*g*-PEG FITC conjugate is anchoring to the silver particles of the silver impregnated fibers. The positively charged end of the PLL conjugate backbone is adsorbing electrostatically onto the negatively charged silver particles. Previous researchers have seen similar results affixing PLL-*g*-PEG ligand to negatively charged surfaces such as PEDOT/PSS [40], TiO_2_ [41] or Nb_2_O_5_ [42]. 

**Figure 6:**
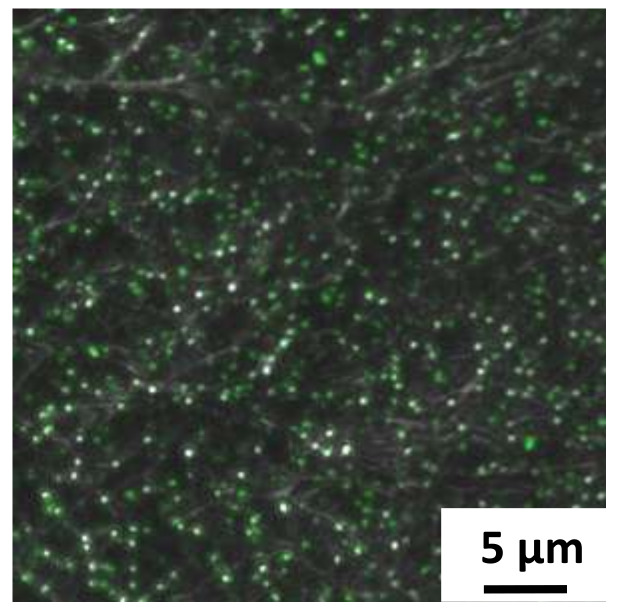
Confocal image of FITC functionalized silver doped carbon nanofibers fiber after being placed in microfluidic channel.

Initial microfluidic tests were done in order to assess whether the electrostatically adsorbed PLL-*g*-PEG FITC was well bound to the silver surface. PLL-*g*-PEG FITC surface functionalized fiber mats were placed in microfluidic channels; PBS buffer solution were pumped through at a rate of 200 μL/min for 50 min. Fiber mat was then removed from the channel and reimaged using the confocal microscope. Figure 6 shows that the green fluorescence of the FITC dye can still be seen. This indicates that the conjugate is well attached to the silver particle on the fiber membrane and can withstand high shear rate within the microfluidic channel environment. 

**Figure 7:**
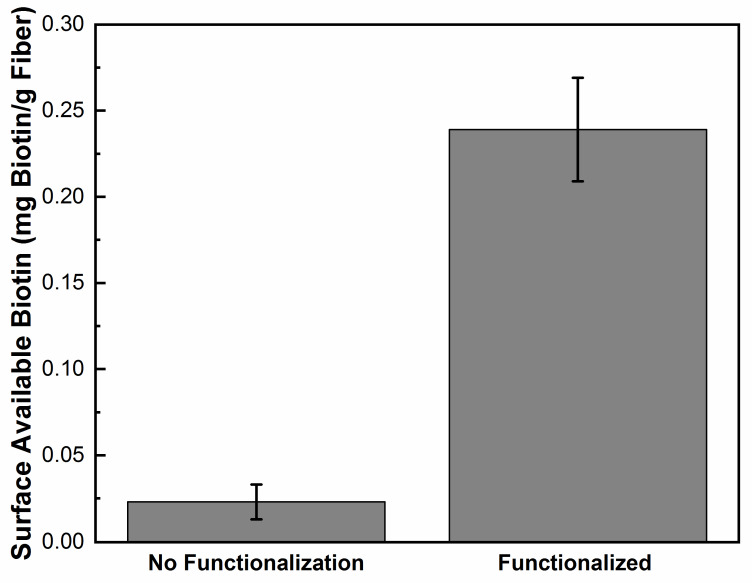
Graph comparing surface available biotin of PLL-*g*-PEG functionalized silver impregnated nanofiber compared to the fiber with no functionalization.

Experiments then proceeded to using the PLL-*g*-PEG biotin conjugate for surface modification of the fiber mat. This conjugate will adsorb onto the silver surface in a similar way to the PLL-*g*-PEG FITC conjugate. After the fibers were placed in the PLL-*g*-PEG biotin solution for 1 h, the mat was washed with buffer solution to remove excess conjugate. A colorimetric assay was used to measure the surface available biotin. Results in Figure 7, show the surface available biotin is 0.24 ± 0.02 mg/g fiber. This is a 10 time increase in surface available biotin compared to silver impregnated nanofibers with no surface modification. 

### 3.1. E. coli Capture

**Figure 8:**
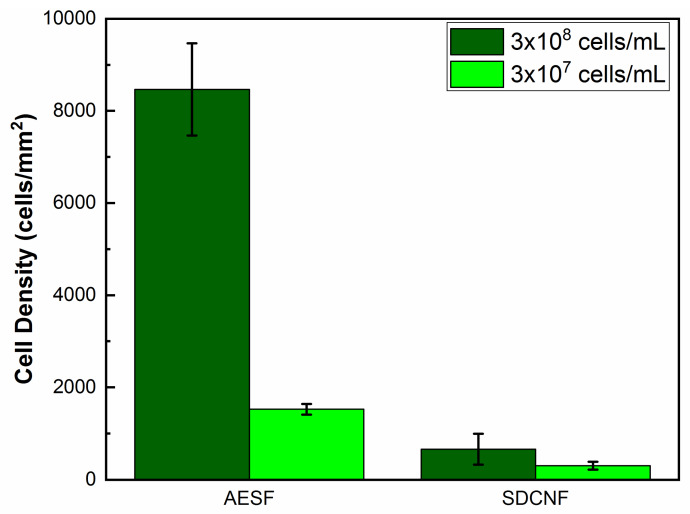
Capture cell density of anti-*E. coli* functionalized fibers and neat silver doped carbon nanofibers.

After confirming the PLL-*g*-PEG biotin conjugate has been attached to fiber mat via the silver nanoparticles, fiber mats are then fully functionalized using streptavidin and biotinylated anti-*E. coli* antibody (Figure 1). Anti-*E. coli* functionalized fibers are then placed in microfluidic channels and *E. coli* solutions of varying concentrations are pumped through. At high concentrations (3 × 10^8^ cells/mL), the fiber mat was able to capture a cell density of 8500 ± 1014 cell/mm^2^ compared to 660 ± 329 cells/mm^2^ compared to the neat silver doped carbon nanofibers (Figure 8). These results show the antibodies are well affixed to the fiber surface and were able to capture more than 12 times the number of bacteria compared to the control fiber mat. 

**Figure 9:**
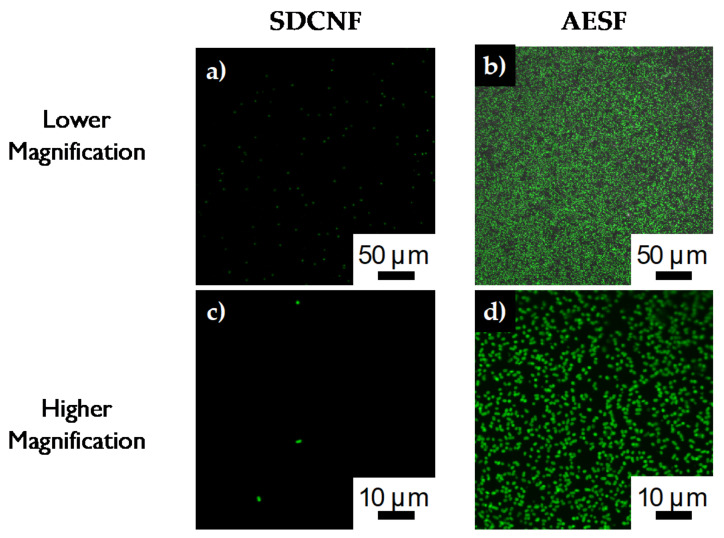
Confocal images of *E. coli* capture on **a**,**c**) SDCNF mats and **b**,**d**) AESF mats at 3 × 10^7^ inlet solution concentration.

Reducing the concentration of *E. coli* in the inlet stream by 10-fold to 3 × 10^7^ cells/mL shows similar trend to the higher concentration experiments. AESF fibers have a capture cell density of approximately 1524 cells/mm^2^ compared to 289 cells/mm^2^ of the SDCNF (Figure 8). This shows a 5.3× increase in the amount of *E. coli* captured in the channels comparing the two nanofiber mat types with 3 × 10^7^ cells/mL inlet stream. Comparing the SDCNF values of the 3 × 10^7^ cells/mL to the 3 × 10^8^ cells/mL system shows a reduction of about half in the amount of *E. coli* captured. This is to be expected since there is less material in the system to interact with the fiber mats and become loosely bound. In comparison, the AESF mats show a 5.5-fold increase in captured cell density comparing the 3 × 10^7^ cells/mL and 3 × 10^8^ cells/mL systems. With the higher concentration system, there is more opportunity for the antibodies present on the surface of the fibers to come into contact with *E. coli* in the channels and bind to them leading to the increase in cell density. With a 10-fold increase in the inlet cell density, one might expect a 10-fold increase in cell capture as opposed to the 6-fold increase observed here. This discrepancy is due to fiber mat saturation and residence time. Figure 9b shows the fiber mat is almost completely covered with FITC-tagged *E. coli* which will fluoresce green under the 488 nm laser line. As more *E. coli* is bound to the fiber surface, not only will it reduce the number of available antibodies, but it will eventually interfere with incoming *E. coli* ability to bind to the surface before being swept away by the incoming flow.

Looking at the confocal images of the fibers after they have been removed from the channels, we can an even better comparison of the capture efficiency of the anti-*E. coli* fibers. Looking at Figure 5b, AESF fibers were completely saturated with bacteria. On the surface of the fiber mat, approximately 1045 ± 189 *E. coli* cells are captured for a 4900 μm^2^ area. Comparing this result to the SDCNF mat, there is 8 ± 3 *E. coli* cells for the same area. As we compare results of the of the cell density results from the fluorescent microscope to the results from the confocal microscope, we notice a discrepancy from the capture efficiency between the functionalized and non-functionalized fibers. Fluorescent microscope data show a 12× increase while the confocal cell density count is 130×. This can be explained by considering when each measurement was conducted. Fluorescent microscope data are gathered comparing the stream entering and exiting the microfluidic channel. While confocal images are taken after the fibers have been rinsed in the channel with buffer solution to remove non-specifically bound bacteria. There is still a large number of bacteria non-specifically loosely bound to the neat fiber mat before rinsing. However, after the rinsing process, the majority of the non-specifically bound *E. coli* is removed from the SDCNF membrane by the buffer solution. On the other hand, the antibodies on the surface of the AESF fibers are able to capture *E. coli* out of solution and bind it to the fiber surface.

**Figure 10:**
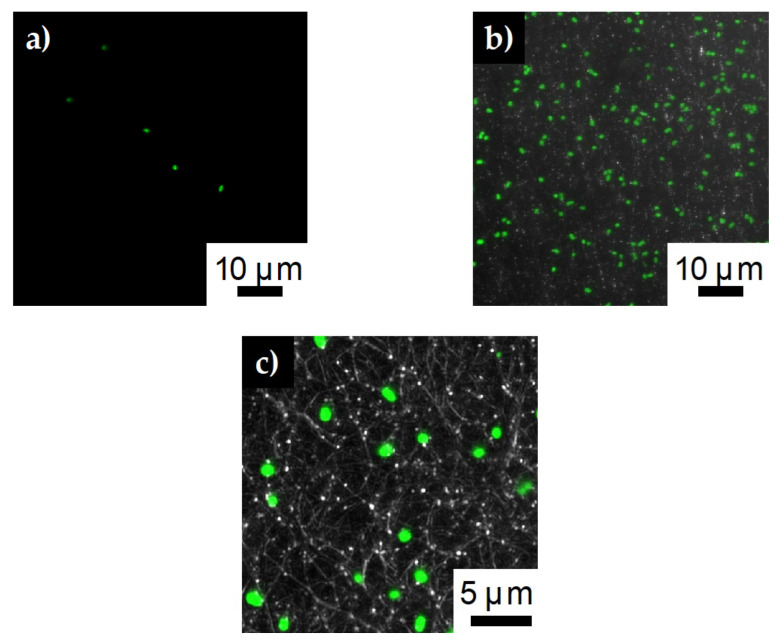
Confocal images of *E. coli* capture on **a**) SDCNF mats and **b**) AESF mats at 3 × 10^7^ inlet solution concentration. **c**) Zoomed in image of AESF fibers to show *E. coli* is bound to areas with silver particles.

Looking at the confocal images for the lower concentration fibers, we again see very little bacteria (5 ± 2 cells) remaining on the SDCNF fiber mats after rinsing (Figure 10a). For the AESF fibers (Figure 10b), we see a capture of about 190 ± 23 *E. coli* cells are captured for the same 4900 μm^2^ area. Though less than the higher concentration tests, this is still a 35-fold increase in the rate of *E. coli* capture compared to the neat fibers at the same concentration. Zooming in further on the AESF (Figure 10c) shows the *E. coli* is lying directly on top of fibers implying the *E. coli* is attached to the fibers and not just resting in the interstitial spaces of the fiber mat. Looking further shows the *E. coli* bacteria lies where the silver particles are present, further indicating the base of the surface functionalization is the silver nanoparticles. Silver particles can be seen along the edges of the bound bacteria indicating those areas are rich in silver particles. Furthermore, showing the large number of immobilized bacteria is not simply bound to the carbon substrate. 

In order to further characterize the capture efficiency of the functionalized substrate, calculations were performed to determine show many cells were capture on the fiber substrate vs. how much bacteria was originally introduced into the channel. Confocal images showed the capture of bacteria onto the substate was uniform and observed on on both sides of the fiber mat. For the high concentration system (input: 3 × 10^8^ cells), the amount of captured cells was calculated to be approximately 5.1 × 10^7^ cells for the AESF substrate and 4.9 × 10^5^ cells for the SDCNF cells. That is a 17% capture efficency for the AESF substrate vs. 0.2% for SDCNF substrate. Functionalization of the silver doped fiber improved the rate of bacteria capture by 100 times. For the lower concentration system (input: 3 × 10^7^ cells), a similar trend is observed. AESF was able to capture approximately 9.6 × 10^6^ cells compared to the 2.4 × 10^5^ capture of the SDCNF fibers. This is a 32% capture efficency for the AESF cells compared to 0.8% of the SDCNF. The lower concentration system appears to have a higher capture efficency compared to the higher concentration system. As noted before, this is due to the functionalized fiber mat nearing its capture threshold for the higher concentration system. Referring back to Figure 9, confocal images show the fiber mat is almost completely covered in the green-flourescent bacteria. In the lower concentration system, the fiber substrate was able to capture more of the input bacteria from the incoming stream since there were more available antibodies and less bacteria collision to inhibit antibody-antigen interaction.

### 3.2. Antibody Selectivity 

**Figure 11:**
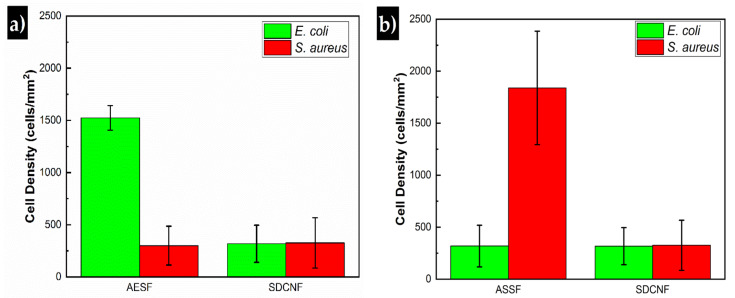
Capture cell density of functionalized fibers and neat silver doped carbon nanofibers using both *E. coli* and *S. aureus.* **a**) Capture cell density of AESF fibers mats vs SDCNF fiber mats after microfluidic studies **b**) Capture cell density of ASSF fibers mats vs SDCNF fiber mats after microfluidic studies.

In order to assess the selectivity of the AESF, Gram negative *E. coli* was substituted with Gram positive *S. aureus* as the bacteria used in the inlet stream while still using anti-*E. coli* antibody to functionalize the silver doped carbon nanofibers. Figure 11a shows that *S. aureus* is captured on the surface of the ASEF fiber mats at the same rate as the SDCNF mats. This indicates the AESF fiber mat is selectively capturing *E. coli* and not simply capturing any particulates present in the inlet stream. These results are in line with previous researchers who have used biotinylated antibodies as the basis of their antigen capture [19,20]. 

Anti-*S. aureus* antibodies were immobilized onto the surface of SDCNF in a similar way to AESF to create anti-*S. aureus* surface functionalized (ASSF) fibers. Experiments done coupling ASSF fibers with Alexa Flour 594 tagged *S. aureus* show a similar rate of bacterium capture for 3 × 10^7^ cells/mL (Figure 11b) inlet stream compared similar results with AESF/*E. coli* system in Figure 11a. In this case, the ASSF fibers were able to capture *S. aureus* at a rate five times higher than that of SDCNF, displaying the functionalized fibers ability to bind a targeted bacterium out of solution. Once *E. coli* is used as the bacterium in microfluidic channel with ASSF fibers, we see that it captures bacteria at a similar rate to SDCNF. Both systems show surface functionalized fibers will bind with target bacterium with high selectivity.

**Figure 12:**
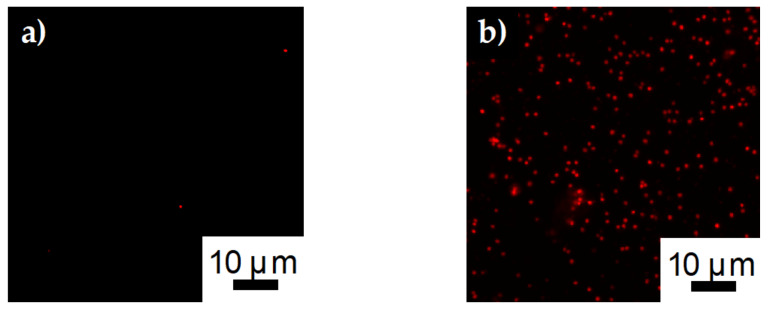
Confocal images of *E. coli* capture on **a**) SDCNF mats and **b**) ASSF mats at 3 × 10^7^ inlet solution concentration.

Confocal images of *S. aureus* bound to SDCNF and ASSF fibers can be seen in Figure 12. The Alexa Flour 594 tagged *S. aureus* fluoresces red under the 561 nm laser line. Similar to Figure 10a,b, there is little bacterium present on the SDCNF fibers compared the ASSF. The number of bacteria captured per 4900 μm^2^ is similar for *E. coli* (190 ± 23) and *S. aureus* (240 ± 46) when comparing surface functionalized fibers. These results show this fiber surface modification technique is tunable for a variety of antigen capture with reproducible results.

## 4. Conclusions

We employed the electrospinning technique to create silver-doped carbon nanofibers. The nanofibers had a large number of silver particles ranging from 125 nm to 225 nm. The negatively charged silver particles serve as the base for further surface functionalization, resulting in the immobilization of anti-*E. coli* antibodies onto the surface of the fiber mat. The AESF fibers were able to capture *E. coli* out of solution at rate 30–80× times the rate of the neat fibers based on the concentration of the inlet bacteria stream. The selectivity of the AESF membranes was also proven by using a different bacterium (*S. aureus*) showing the AESF captured this bacterium at the same rate as SDCNF. 

## References

[1] Godfray H.C.J., Beddington J.R., Crute I.R., Haddad L., Lawrence D., Muir J.F., Pretty J., Robinson S., Thomas S.M., Toulmin C. (2010). Food Security: The Challenge of Feeding 9 Billion People. Science.

[2] King T., Cole M., Farber J.M., Eisenbrand G., Zabaras D., Fox E.M., Hill J.P. (2017). Food safety for food security: Relationship between global megatrends and developments in food safety. Trends Food Sci. Technol..

[3] Omari R., Frempong G.K., Arthur W. (2018). Public perceptions and worry about food safety hazards and risks in Ghana. Food Control.

[4] Chen J., Park B. (2018). Label-free screening of foodborne Salmonella using surface plasmon resonance imaging. Anal. Bioanal. Chem..

[5] Scallan E., Hoekstra R.M., Angulo F.J., Tauxe R.V., Widdowson M.-A., Roy S.L., Jones J.L., Griffin P.M. (2011). Foodborne illness acquired in the United States—Major pathogens. Emerg. Infect. Dis..

[6] Scallan E., Griffin P.M., Angulo F.J., Tauxe R.V., Hoekstra R.M. (2011). Foodborne illness acquired in the United States—Unspecified agents. Emerg. Infect. Dis..

[7] Zheng L., Cai G., Wang S., Liao M., Li Y., Lin J. (2019). A microfluidic colorimetric biosensor for rapid detection of Escherichia coli O157:H7 using gold nanoparticle aggregation and smart phone imaging. Biosens. Bioelectron..

[8] Wu X., Wang W., Liu L., Kuang H., Xu C. (2015). Monoclonal antibody-based cross-reactive sandwich ELISA for the detection of Salmonella spp. in milk samples. Anal. Methods.

[9] Beauchamp S., D’Auria S., Pennacchio A., Lacroix M. (2012). A new competitive fluorescence immunoassay for detection of Listeria monocytogenes. Anal. Methods.

[10] Liébana S., Brandão D., Alegret S., Pividori M.I. (2014). Electrochemical immunosensors, genosensors and phagosensors for Salmonella detection. Anal. Methods.

[11] Wang W., Feng M., Kong D., Liu L., Song S., Xu C. (2015). Development of an immunochromatographic strip for the rapid detection of Pseudomonas syringae pv. maculicola in broccoli and radish seeds. Food Agric. Immunol..

[12] Montserrat M., Sanz D., Juan T., Herrero A., Sánchez L., Calvo M., Pérez M.D. (2015). Detection of peanut (Arachis hypogaea) allergens in processed foods by immunoassay: Influence of selected target protein and ELISA format applied. Food Control.

[13] Wang S., Inci F., Chaunzwa T.L., Ramanujam A., Vasudevan A., Subramanian S., Chi Fai Ip A., Sridharan B., Gurkan U.A., Demirci U. (2012). Portable microfluidic chip for detection of Escherichia coli in produce and blood. Int. J. Nanomed..

[14] Jalali M., AbdelFatah T., Mahshid S.S., Labib M., Perumal A.S., Mahshid S. (2018). A Hierarchical 3D nanostructured microfluidic device for sensitive detection of pathogenic bacteria. Small.

[15] Wang M., Xiao Y., Lin L., Zhu X., Du L., Shi X. (2018). A microfluidic chip integrated with hyaluronic acid-functionalized electrospun chitosan nanofibers for specific capture and nondestructive release of CD44-overexpressing circulating tumor cells. Bioconjugate Chem..

[16] Liu H., Yu X., Cai B., You S., He Z., Huang Q., Rao L., Li S., Liu C., Sun W. (2015). Capture and release of cancer cells using electrospun etchable MnO2 nanofibers integrated in microchannels. Appl. Phys. Lett..

[17] Matlock-Colangelo L., Coon B., Pitner C.L., Frey M.W., Baeumner A.J. (2016). Functionalized electrospun poly(vinyl alcohol) nanofibers for on-chip concentration of E. coli cells. Anal. Bioanal. Chem..

[18] Zhang N., Deng Y., Tai Q., Cheng B., Zhao L., Shen Q., He R., Hong L., Liu W., Guo S. (2012). Electrospun TiO2 nanofiber-based cell capture assay for detecting circulating tumor cells from colorectal and gastric cancer patients. Adv. Mater..

[19] Yu C.-C., Ho B.-C., Juang R.-S., Hsiao Y.-S., Naidu R.V.R., Kuo C.-W., You Y.-W., Shyue J.-J., Fang J.-T., Chen P. (2017). Poly(3,4-ethylenedioxythiophene)-based nanofiber mats as an organic bioelectronic platform for programming multiple capture/release cycles of circulating tumor cells. ACS Appl. Mater. Interfaces.

[20] Hsiao Y.-S., Ho B.-C., Yan H.-X., Kuo C.-W., Chueh D.-Y., Yu H., Chen P. (2015). Integrated 3D conducting polymer-based bioelectronics for capture and release of circulating tumor cells. J. Mater. Chem. B.

[21] Zhao Y., Fan Z., Shen M., Shi X. (2015). Hyaluronic acid-functionalized electrospun polyvinyl alcohol/polyethyleneimine nanofibers for cancer cell capture applications. Adv. Mater. Interfaces.

[22] Xiao Y., Lin L., Shen M., Shi X. (2019). Design of DNA aptamer-functionalized magnetic short nanofibers for efficient capture and release of circulating tumor cells. Bioconjugate Chem..

[23] Hou S., Zhao L., Shen Q., Yu J., Ng C., Kong X., Wu D., Song M., Shi X., Xu X. (2013). Polymer nanofiber-embedded microchips for detection, isolation, and molecular analysis of single circulating melanoma cells. Angew. Chem. Int. Ed..

[24] Jin S., Dai M., Ye B., Nugen S.R. (2013). Development of a capillary flow microfluidic Escherichia coli biosensor with on-chip reagent delivery using water-soluble nanofibers. Microsyst. Technol..

[25] Luo Y., Nartker S., Miller H., Hochhalter D., Wiederoder M., Wiederoder S., Setterington E., Drzal L.T., Alocilja E.C. (2010). Surface functionalization of electrospun nanofibers for detecting E. coli O157:H7 and BVDV cells in a direct-charge transfer biosensor. Biosens. Bioelectron..

[26] Li C.Y., Wan Y.Z., Wang J., Wang Y.L., Jiang X.Q., Han L.M. (1998). Antibacterial pitch-based activated carbon fiber supporting silver. Carbon.

[27] Kong H., Jang J. (2008). Antibacterial properties of novel poly(methyl methacrylate) nanofiber containing silver nanoparticles. Langmuir.

[28] Song J., Kang H., Lee C., Hwang S.H., Jang J. (2012). Aqueous synthesis of silver nanoparticle embedded cationic polymer nanofibers and their antibacterial activity. ACS Appl. Mater. Interfaces.

[29] Song K., Wu Q., Zhang Z., Ren S., Lei T., Negulescu I.I., Zhang Q. (2015). Porous carbon nanofibers from electrospun biomass tar/polyacrylonitrile/silver hybrids as antimicrobial materials. ACS Appl. Mater. Interfaces.

[30] Li D., Frey M.W., Vynias D., Baeumner A.J. (2007). Availability of biotin incorporated in electrospun PLA fibers for streptavidin binding. Polymer.

[31] González E., Shepherd L.M., Saunders L., Frey M.W. (2016). Surface functional poly(lactic acid) electrospun nanofibers for biosensor applications. Materials.

[32] Ren Y., Ray S., Liu Y. (2019). Reconfigurable acrylic-tape hybrid microfluidics. Sci. Rep..

[33] Nath P., Fung D., Kunde Y.A., Zeytun A., Branch B., Goddard G. (2010). Rapid prototyping of robust and versatile microfluidic components using adhesive transfer tapes. Lab. Chip..

[34] Patko D., Mártonfalvi Z., Kovacs B., Vonderviszt F., Kellermayer M., Horvath R. (2014). Microfluidic channels laser-cut in thin double-sided tapes: Cost-effective biocompatible fluidics in minutes from design to final integration with optical biochips. Sens. Actuators B Chem..

[35] Han Z., Jiang X. (2019). Microfluidic Synthesis of Functional Nanoparticles. Nanotechnology and Microfluidics.

[36] Oza T.M., Oza V.T., Thaker R.H. (1955). The thermal decomposition of silver nitrite. J. Chem. Soc..

[37] Abdo H.S., Khalil K.A., Al-Deyab S.S., Altaleb H., Sherif E.-S.M. (2013). Antibacterial effect of carbon nanofibers containing Ag nanoparticles. Fibers Polym..

[38] Barua B., Saha M.C. (2018). Influence of humidity, temperature, and annealing on microstructure and tensile properties of electrospun polyacrylonitrile nanofibers. Polym. Eng. Sci..

[39] Zhmayev Y., Pinge S., Shoorideh G., Shebert G.L., Kaur P., Liu H., Joo Y.L. (2016). Controlling the Placement of spherical nanoparticles in electrically driven polymer jets and its application to li-ion battery anodes. Small.

[40] Hong W.Y., Jeon S.H., Lee E.S., Cho Y. (2014). An integrated multifunctional platform based on biotin-doped conducting polymer nanowires for cell capture, release, and electrochemical sensing. Biomaterials.

[41] Kenausis G.L., Vörös J., Elbert D.L., Huang N., Hofer R., Ruiz-Taylor L., Textor M., Hubbell J.A., Spencer N.D. (2000). Poly(l-lysine)-g-Poly(ethylene glycol) layers on metal oxide surfaces: attachment mechanism and effects of polymer architecture on resistance to protein adsorption. J. Phys. Chem. B.

[42] Huang N.-P., Michel R., Voros J., Textor M., Hofer R., Rossi A., Elbert D.L., Hubbell J.A., Spencer N.D. (2001). Poly(l-lysine)-g-poly(ethylene glycol) layers on metal oxide surfaces: surface-analytical characterization and resistance to serum and fibrinogen adsorption. Langmuir.

